# The *Agaricus blazei*-Based Mushroom Extract, Andosan^™^, Protects against Intestinal Tumorigenesis in the A/J Min/+ Mouse

**DOI:** 10.1371/journal.pone.0167754

**Published:** 2016-12-21

**Authors:** Geir Hetland, Dag M. Eide, Jon M. Tangen, Mads H. Haugen, Mohammad R. Mirlashari, Jan E. Paulsen

**Affiliations:** 1 Department of Immunology, Oslo University Hospital, Oslo, Norway; 2 Institute of Clinical Medicine, University of Oslo, Oslo, Norway; 3 Department of Chemicals and Radiation, Norwegian Institute of Public Health, Oslo, Norway; 4 Department of Acute Medicine & National CBRNE Medical and Advisory Centre–Norway, Oslo University Hospital, Oslo, Norway; 5 Department of Tumor Biology, Institute for Cancer Research, Oslo University Hospital – The Norwegian Radium Hospital, Oslo, Norway; 6 Norwegian University of Life Sciences, Department of Food Safety and Infection Biology, Oslo, Norway; Institute of Pathology, GERMANY

## Abstract

**Background:**

The novel A/J Min/+ mouse, which is a model for human Familial Adenomatous Polyposis (FAP), develops spontaneously multiple adenocarcinomas in the colon as well as in the small intestine. *Agaricus blazei* Murill (AbM) is an edible *Basidiomycetes* mushroom that has been used in traditional medicine against cancer and other diseases. The mushroom contains immunomodulating β-glucans and is shown to have antitumor effects in murine cancer models. Andosan^™^ is a water extract based on AbM (82%), but it also contains the medicinal *Basidiomycetes* mushrooms *Hericeum erinaceus* and *Grifola frondosa*.

**Methods and findings:**

Tap water with 10% Andosan^™^ was provided as the only drinking water for 15 or 22 weeks to A/J Min/+ mice and A/J wild-type mice (one single-nucleotide polymorphism (SNP) difference), which then were exsanguinated and their intestines preserved in formaldehyde and the serum frozen. The intestines were examined blindly by microscopy and also stained for the tumor-associated protease, legumain. Serum cytokines (pro- and anti-inflammatory, Th1-, Th2 -and Th17 type) were measured by Luminex multiplex analysis. Andosan^™^ treated A/J Min/+ mice had a significantly lower number of adenocarcinomas in the intestines, as well as a 60% significantly reduced intestinal tumor load (number of tumors x size) compared to control. There was also reduced legumain expression in intestines from Andosan^™^ treated animals. Moreover, Andosan^™^ had a significant cytotoxic effect correlating with apoptosis on the human cancer colon cell line, Caco-2, in vitro. When examining serum from both A/J Min/+ and wild type mice, there was a significant increase in anti-tumor Th1 type and pro-inflammatory cytokines in the Andosan^™^ treated mice.

**Conclusions:**

The results from this mouse model for colorectal cancer shows significant protection of orally administered Andosan^™^ against development of intestinal cancer. This is supported by the finding of less legumain in intestines of Andosan^™^ treated mice and increased systemic Th1 cytokine response. The mechanism is probably both immuno-modulatory and growth inhibition of tumor cells by induction of apoptosis.

## Introduction

*Agaricus blazei* Murill (AbM) is an edible medicinal mushroom of the family, *Basidiomycetes*, and a close relative to *Agaricus bisporus*, the champignon. AbM was first described in 1893 and it is also known as *Agaricus subrufesence*, *A*. *rufotegulis* and recently as *A*. *brasiliensis* because of its origin in a rain forest area near Piedade, Brazil [1]. According to legend, the frequency of geriatric diseases, including cancer, was lower there than in neighboring areas due to the local high intake of AbM as food in Piedade. Since the 1960-ies Japanese researchers have detected immuno-modulating and antitumor properties of AbM in studies in the mouse [2,3]. *Hericium erinaceus* and *Grifola frondosa* are two other edible *Basidiomycetes* mushrooms used in traditional Eastern medicine that have similar antitumor effects [4,5].

In collaboration with Shinshu Agricultural University, Nagano, Japan, a strain of AbM was chosen that had optimal properties both with regard to assumed health effects and cultivation ability. It was mixed with 15% of *H*. *erinaceus* and 3% of *G*. *frondosa* to obtain a more potent product. In 2004 this AbM-based mixed *Basidiomycetes* mushroom extract was found to be the only one among other Japanese AbM extracts tested blindly in a pneumococcal sepsis mouse model at the Norwegian Institute of Public Health, Oslo, that significantly reduced bacteremia and increased the animals’ survival rate [6]. It was later trade-marked Andosan^™^ and chosen for further studies, which showed that it also protected against Gram-negative sepsis [7] and allergy [8] in other mouse models. These effects together with the antitumor property of the mushrooms contained in Andosan^™^, is probably foremost due to the immuno-modulatory relative shift induced by the mushroom, from a Th2 to a predominant Th1 response [9, 8]. In human studies we have found that Andosan^™^ induced increased expression of genes related to cancer defence in peripheral blood leukocytes (cell signaling and cycling, and transcriptional regulation) [10], and it also proved to have anti-inflammatory properties in IBD patients and healthy individuals without any pathological findings in blood samples or clinical side effects [11, 12]. In a recent placebo-controlled clinical study, Andosan^™^ was given orally as adjuvant therapy for patients with multiple myeloma undergoing high-dose chemotherapy and bone marrow transplantation and found to have immunomodulatory and anti-inflammatory effects [13]. Clinically, trends for a longer median time to next treatment and shorter antibiotics use were noted in the mushroom extract group. The assumed role of AbM in immune system modulation and disease control is reviewed in Hetland et al 2011 [14].

Previously, β-glucan polysaccharide [3] and ergosterol containing lipid [15] isolated from AbM, have been shown to have in vivo antitumor activity in transplantable tumor-bearing mouse models. Later, chemically induced carcinogenesis has been used as experimental tumor models in rodents for both hepatocarcinogenesis and colon carcinogenesis studies [16,17] on possible beneficial effects of AbM extracts. The former study demonstrated hepatoprotective effect of orally administered *A*. *blazei* extract [16], and the latter [17] showed tendency to reduced dysplastic aberrant crypt formation in colon but no difference in colon tumor incidence after *A*. *blazei* ingestion [17].

Colorectal cancer is the 4th most frequent type of cancer in Western societies, but the 2nd deadliest after lung cancer [18]. There is increasing evidence for a link between inflammation and colorectal cancer [19]. Approximately 14% of colon cancer has a familial background, such as for the inflammatory bowel disease, ulcerative colitis, and 1% is caused by familial adenomatous polyposis (FAP) [18]. The multiple intestinal neoplasia (Min/+) mouse is frequently used as model for human FAP and colorectal cancer because it is heterozygous for a mutation in the tumor suppressor gene APC, which leads to the formation of numerous intestinal adenomas [20,21]. Complete somatic inactivation of APC in discrete crypts of the intestinal epithelium appears to be the initial event of the tumorigenesis in Min/+ mice, human FAP and in the majority of sporadic colorectal cancer in humans [22]. In contrast to human FAP, conventional C57 BL/6J Min/+ mice develop tumors predominantly in the small intestine [23,24,25,26]. Therefore, the novel Min/+ mouse on the A/J genetic background provides a better model for colon cancer because these mice spontaneously develop numerous colonic adenomas that eventually progress to carcinomas in old individuals [27].

Legumain is a tumor-associated proteolytic enzyme (asparaginyl endopeptidase) that is expressed in kidney, placenta, and spleen [28] and important for normal kidney function [29]. High levels of the protease legumain have been detected in solid tumors and associated with increased tumor invasion and metastasis [30,31]. The protease has been detected on tumor cell surface and in tumor microenvironment [32], where it has been shown to destroy extracellular matrix by degrading its major component, fibronectin [33]. Moreover, legumain is found on tumor associated macrophages, which are important for tumor development and metastasis [34]. Legumain is highly expressed in colorectal cancer cell lines and associated with poor outcome in colon cancer [35]. Recently, we have shown that Andosan^™^ reduced the activity of legumain in rat macrophages [36].

In the present study we have investigated whether the AbM-based Andosan^™^ extract had any influence on the development of adenomatous tumors in small intestines and colon/rectum of A/J *Min/+* mice when added to the drink water, and whether it affected intestinal expression of the tumor-associated and metastasis-promoting protease, legumain. Moreover, we measured serum cytokine levels in the animals and examined putative cytotoxic effect of the mushroom extract on the human colon cancer cell line, Caco-2.

## Materials and Methods

### Reagents and cell line

Andosan^™^ is a mixed *Basidiomycetes* mushroom water extract of the mycelium of AbM (82.4%), *Hericeum erinaceus* (14.7%) and *Grifola frondosa* (2.9%) produced and heat-(124°C for 1 hour) sterilized by ACE Co Ltd, Gifu-ken, Japan, and GMP-certified by Meiji Co. Ltd, Japan, and contained and stored in sterile dark-glassed bottles at room temperature until use. The extract with a final concentration of 340 g/l was imported to Norway as food (mushroom juice) and provided by the company Immunopharma AS (organization no. 994924273), Oslo, Norway. The LPS content of Andosan^™^ was found be <0.5 pg / ml, using the Limulus amebocyte lysate test (COAMATIC Chromo-LAL; Chromogenix, Falmouth, MA, USA). The results from tests for heavy metals were conformable with Japanese regulations for health foods and potential radioactivity in the extract was not detected.

### Caco-2 cells cultures and *in vitro* cytotoxicity

The human epithelial colorectal adenocarcinoma cells (Caco-2) were obtained from American Type Culture Collection (ATCC^®^ HTB-37^™^, no:HTB-37) and were grown in Eagle’s Minimum Essential Medium (EMEM, cat no. 30–2003, ATCC, USA) supplemented with 10% fetal bovine serum (FBS, cat no. 30–2020, ATCC, USA) and 1% antibiotic mix (Sigma Aldrich, cat. no. A5955) and maintained in a humidified atmosphere with 5% CO_2_ at 37°C in an incubator (Forma Series II Water Jacket 3111, Thermo Scientific) and the media were changed twice a week. Cytotoxicity experiments were conducted using cells with less than 5 passages. For cytotoxicity assay, 5 x 10^3^cells/cm^2^ were seeded onto 25 cm^2^ culture flasks and allowed to attach overnight. The medium was then replaced with new medium containing various concentrations of Andosan (0.5, 1.0, 2.5 and 5.0%) or PBS (control). After 96 hours cells were washed 3 times with PBS (Sigma, D8537) and the cells were harvested by using Trypsin-EDTA solution (ATCC, cat. no. 30–210). The total number and percent viable cells were counted by NucleoCounter using the NucleoCassette kit (Chemometec, Allerød, Denmark) according to the manufacture's manual.

### Quantification of cellular apoptosis by flow cytometry

Following the examination of the effect of Andosan^™^ on cell growth, the effect of Andosan^™^ on cell apoptosis was determined and the percentage of apoptotic cells was quantified using the annexin V. The annexin V-binding assays were performed according to manufacturer's protocol (BD PharMingen). Briefly, cultured cells (1 × 10^6^) were collected, washed twice with cold PBS and resuspended in 1 ml binding buffer. Then 5 μl of Fluorescein isothiocyanate (FITC)-conjugated annexin V (Cat. No. 556419, BD Pharminogen, San Jose, CA, USA) and 7-aminoactinomycin (7-AAD, cat, No. 51-68981E, BD Pharminogen, San Jose, CA, USA). were added to 100 μl of cell suspension and incubated for 15 minutes at room temperature in the dark. Finally, 400 μl of binding buffer (Cat. No. 556454, BD Biosciences, USA) was added to samples and analyzed by using the Gallios flow cytometer as soon as possible (within 1 hour).

### Mouse breeding, experimental design, and scoring of intestinal lesions

This study was carried out in strict accordance with the laws and regulations for animal experiments in Norway and was approved by the National Experimental Animal Board in Norway. All mice (originally purchased from The Jackson Laboratory, Bar Harbor, ME) were bred at the animal facility of Norwegian Institute of Public Health. The *Min/+* trait from C57BL/6J *Min/+* mice was transferred to A/J mice by backcrossing for more than 12 generations.

For the purpose of this experiment, A/J *Min*/+ males/females were mated with A/J +/+ (wild type) females/males, with one or two females and one male housed in each disposable plastic cage (Innovive, San Diego, CA, USA) on aspen chip bedding in paper bags for enrichment (Nestpack, Datesand, Manchester, UK). The animal room was maintained on a 12-hour light/dark cycle, with controlled humidity (55 ± 5%) and temperature (20–24°C). Tap water and feed was available *ad libitum*. When 3–4 weeks old, the *Min/+* and wild type pups were identified by an allele-specific PCR-assay, as described previously [37], individually ear punched, and each litter was separated in male and female cages. In total, A/J *Min/+* mice (N = 46) and wild type mice (N = 30) from 28 litters were included. All animals were fed a standard rodent diet RM1 (Special Diet Services, Witham, UK) for the duration of the study. The communal tap water was routinely monitored for microbiological quality and ion content, supplied to the mice in disposable plastic bottles (Innovive). The cages were randomly divided in two groups, either receiving were given 10% of Andosan^™^ *Agaricus* bM extract in tap water, or tap water only (controls), for 15 or 22 weeks and killed with CO_2_ and exsanguinated for serum (1900G, 10min, 15°C) that was frozen (-80°C).

The small intestine and the colon were removed and rinsed in ice-cold PBS before they were longitudinally incised and fixed flat between wet (PBS) filter papers for a minimum of 24 hours in 10% neutral buffered formalin. After a 5 second stain in 0.2% methylene blue (George T. Gurr Ltd., London, United Kingdom) dissolved in 10% neutrally buffered formalin. The intestinal surface was examined by transillumination in the inverse light microscope equipped with an eyepiece graticule to determine the number (#), size (mm^2^) and tumor load (mm^2^), defined as the sum of the area of all tumors (# tumors x size). A colonic tumor was defined as a lesion with >30 aberrant crypts.

### Legumain immunofluorescence staining of intestines

Swiss-rolls were prepared [27] by longitudinal opening of the intestine followed by rolling up along the same axis with the lumen facing inwards and embedding the rolls into parrafin blocks. Immunofluorescence staining was performed on deparaffinized sections of swiss-rolled intestines from the A/J Min/+ mice using a primary antibody against legumain (R&D Systems; AF2199) and secondary antibody conjugated with Alexa 488 (Jackson ImmunoResearch). Stained sections were analyzed by fluorescent microscopy (Olympus IX-81) using the corresponding software (Cell^P).

### Cytokine measurement

Serum cytokine measurements were performed using the multiplex bead-based sandwich immunoassay Bio-Plex xMAP technology (Bio-Rad, Austin, TX, USA) with a Luminex IS 100 instrument (Bio-Rad, Hercules, CA, USA) and Bio-Plex Manager (version 6.0.1) software for analysis of 12 different cytokines (IL-1β, IL-2, IL-4, IL-5, IL-6, IL-10, IL-12p70, IL-17, GM-CSF, MCP-1, TNFα, and IFNγ).

### Statistical analysis

Cytokine values and tumor numbers were dependent variables in ANCOVAs (JMP pro, SAS Institute, Cary, NC, USA) where the effects of genotype (*Min* or wildtype) and treatment group (Andosan or water) were evaluated together with age of the pups and sex as covariates. Using the minimum AIC criterion, genotype, age and sex was removed from the model and only the effect of treatment is reported.

The random effect of litter within treatment (N = 28) was also evaluated [38] and found significant, but not taken into account because of the high number of litters within each treatment group would increase the risk of type II error for the effect of treatment.

Transformations of data did not improve the heteroscedasticity of some data, and non-parametric tests resulted in only minor changes in p-values. Thus, we used the same analysis for all responses. No correction of p-values is done for the number of tests applied (e.g., Bonferroni), even if some of the responses are correlated.

### Ethical considerations

The investigation was approved by the local ethical committee, implementing national laws for animal studies.

## Results

### Cytotoxic effect of Andosan^™^ in vitro on human cancer coli cell line

**Fig 1:**
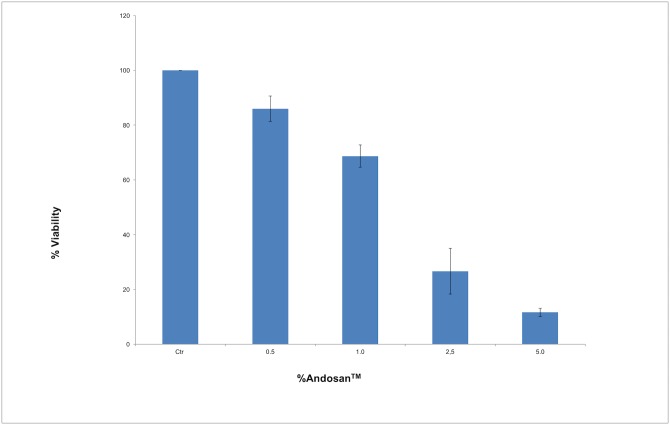
Effect of Andosan^™^ on the proliferation of Caco-2 cell line *in vitro*. Various concentrations of Andosan^™^ (0.5%–5.0%) were added to the culture medium and viability was assessed after 96 h. Results are expressed as mean ± SD in percentage of proliferation relative Caco-2 cells cultured without Andosan^™^ (= 100%) and represent 3 independent experiments. Ctr: control.

**Fig 2:**
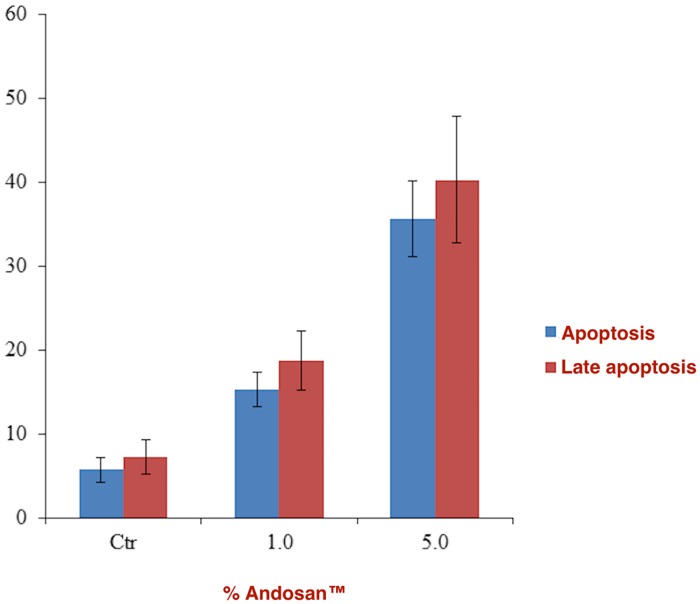
Andosan^™^ induces apoptosis in Caco-2 cells. Caco-2 cells were treated for 96 h with 1.0% or 5.0% Andosan^™^. Binding of annexin V was used as marker for apoptosis and 7-AAD as marker for late apoptosis (necrosis). The results are expressed as mean ± SD and represent 3 independent experiments. Ctr: control.

In vitro antitumor effect of Andosan^™^ was examined in cultures of Caco-2 cells after 96h. Andosan^™^ reduced Caco-2 cell viability in a concentration (0.5–5.0%) dependent manner (Spearman’s correlation coefficient rho ρ = -0.986, p<0.001 (Fig 1). Whereas the concentration of 5% of Andosan^™^ induced killing of near 90% of the Caco-2 cells (two-tailed t-test, p<0.001, even 0.5% of Andosan^™^ had a significant, albeit low (14%), cytotoxic effect (two-tailed t-test, p = 0.006). We next assessed whether the growth inhibitory effect of Andosan^™^ on Caco-2 cells was correlated with increased apoptosis. After treatment with 1 and 5.0% for 96 h, cellular apoptosis was measured by flow cytometry using the annexin V/7-AAD dual cell staining. Treatment with Andosan^™^ 1.0% and 5.0% for 96 hours increased the population of early apoptotic cells (7-AAD-negative and annexin V-positive cells) from 5.7% ± 1.5% in untreated cells to 15.3% ±2.1 for Andosan^™^ 1% and 35.6% ± 4.5% for Andosan^™^ 5.0% treated cells (p<0.01) (Fig 2). The population of late apoptotic cells (7-AAD-positive and annexin V positive cells) increased from 7.3% ± 2.1% for untreated cells to 35.6 ± 4.5 for Andosan^™^ 1.0% and 39.7% ± 7.6% for Andosan^™^ 5.0% treated cells (p<0.01). This result suggests that Andosan^™^ induced growth inhibition of Caco-2 cells, at least in part, by induction of apoptosis.

### Antitumor effect of Andosan^™^ in A/J *Min/+* mice

**Fig 3:**
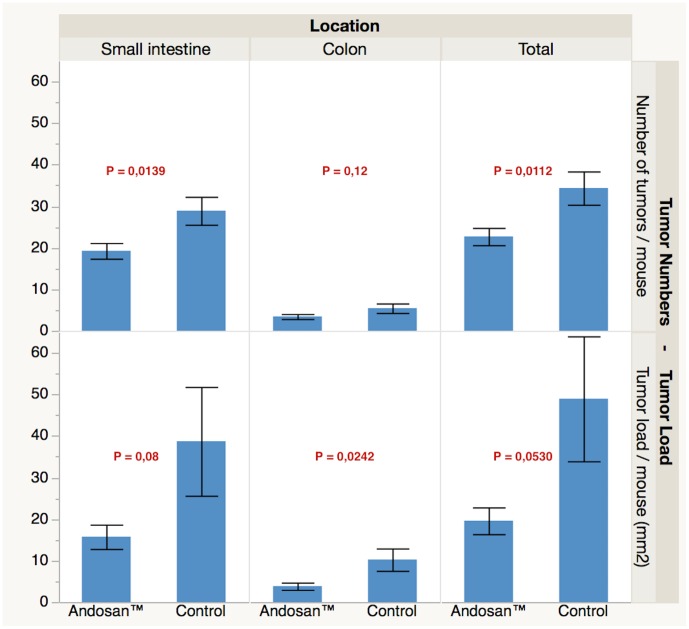
The effect of Andosan^™^ on the development of tumors in small intestine and colon in A/J Min/+ mice. Andosan^™^ (10%) was added or not (control) in drink water to A/J Min/+ mice (n = 46) for 22 weeks, when the animals were killed and their intestines were examined by microscopy. Both the number of tumors (top panel) and the tumor load (# tumors x size) was lower in the Andosan^™^ relative to the control group.

In A/J *Min/+* mice given 10% of Andosan^™^ in the drink (tap) water for 22 weeks, in average 23 tumors/mouse were found in their intestines by microscopy of the formalin preserved tissue. This was statistically significantly (p = 0.021) fewer tumors than the average 34 tumors/mouse counted in intestines of such mice given only ordinary drink (tap) water (Fig 3). The microscopy was done in a blinded fashion in such a way that the treatment group of the individual mouse examined, was unknown to the pathologist. When the size of the tumors was noted and multiplied with number of tumors, there was an approximately 60% significant reduction in the tumor load in both small intestines (p<0.001) and colon/rectum (p = 0.024) of the Andosan^™^ treated mice relative to the tumor load in the control animals (Fig 3). Similar but less pronounced findings were done in intestines of animals treated for 15 weeks with or without Andosan^™^ (data not shown). Also intestines of wild type mice were examined and found not to contain tumors. Since there was no difference in body weight or cecum weight between the groups (not shown), Andosan^™^ in drink water did not affect water or feed intake or intestinal bacterial load and should therefore not bias the results.

### Effect on legumain expression in intestines

**Fig 4:**
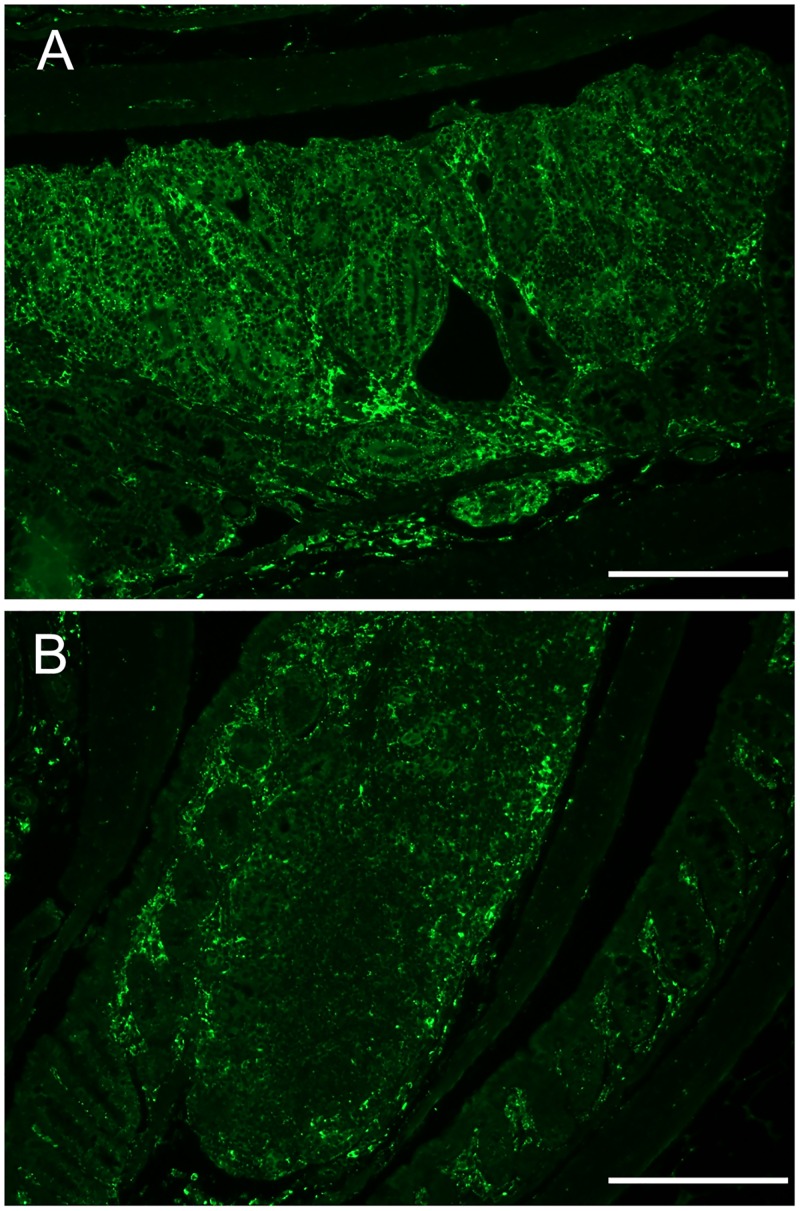
Representative sections showing overall higher expression of legumain (diffuse yellowish fluorescence staining) in the untreated (A) versus the Andosan^™^-treated (B) intestine. Notably, legumain expression was higher in tumor tissue seen in untreated animals. Scale bars represent 200 μm.

In swiss-roll sections of intestines immunofluorescently stained for legumain a strong expression of this metastasis-promoting protease was seen in the spontaneous tumors of untreated animals. Furthermore, overall less expression was observed in the intestine from the Andosan^™^ treated compared with the untreated (Fig 4) animals. However, because no tumors could be identified in sections from the treated animal, a putative difference in legumain expression within tumors could not be shown.

### Serum cytokine profiles in A/J Min/+ and A/J wild-type mice

Sera of both A/J Min/+ mice and wild-type controls that had been sacrificed by exsanguination, were subjected to Luminex multi(12-)plex analysis for Th1, Th2, Th17, pro- and anti-inflammatory cytokines. For Andosan^™^-treated compared with untreated animals, there was a significant increase in Th1 type cytokine IL-12p70 and in pro-inflammatory cytokines IL-1β, MCP-1 and TNFα (Table 1). However, Th2 and Th17 type cytokine responses were not affected.

**Table 1:** The Effect of Treatment with Andosan on Cytokine Values and Tumor Counts.

Response	Estimate*	P-value
IL-1β	36,34	***0*,*0013***
IL-2	2,14	0,52
IL-4	-2,10	0,66
IL-5	-3,19	0,44
IL-6	-4,58	0,41
IL-10	8,48	*0*,*06*
IL-17A	-5,28	0,83
GM-CSF	14,59	***0*,*0039***
IFN-y	-0,55	0,94
MCP-1	51,25	*0*,*03*
TNFα	92,34	***0*,*0013***
IL-12p70	46,70	***0*,*0018***
Number of small intestinal tumors	-4,83	***0*,*0139***
Number of colon tumors	-1,00	0,12
Total tumor number	-5,83	***0*,*0112***
Tumor load small intestine	-11,49	0,08
Tumor load colon	-3,20	***0*,*0242***
Total tumor load	-14,68	***0*,*0530***

* Positive values = Andosan treated group means are higher

## Discussion

Our data show that the mixed *Basidiomycetes* mushroom extract Andosan^™^ given orally, protects against development of intestinal cancer in the colorectal cancer model, A/J *Min/+* mice. This is supported by the finding of i) Lower levels of the tumor-associated protease, legumain, in intestines of the Andosan^™^ treated A/J *Min/+* mice, ii) Higher serum levels of Th1 type cytokine, IL-12p70 in the treated A/J *Min/+* and wild-type mice, and iii) Strong, dose-dependent cytotoxic effect induced in vitro by Andosan^™^ on the human cancer colon cell line, Caco-2. Previously, another AbM extract has been shown to have cytotoxic effect on yet another human cancer colon cell line, HT-29, in addition to eight other human cancer cell lines [17]. Recently, we have found anti-proliferative effect of Andosan^™^ on a murine myeloma cell line [13]. Of the two additional mushrooms contained in Andosan^™^, *Hericium erinaceus*, which comprises ~15% of the mixed mushroom extract, has been shown to specifically inhibit metastasis of colon cancer in a transplanted mouse model [4]. Also the other *Basidiomycetes* mushroom, *Grifola frondosa*, comprising ~3% of Andosan^™^, has antitumor effects in mice [5, 39]. Moreover, AbM extracts have been shown to exibit apoptotic effect on leukemia cells [40], which was the mechanism of death of Caco-2 cells incubated with Andosan^™^ as well.

Within tumors, legumain is produced by several cell types, including tumor-associated macrophages. While this metastasis-promoting protease was observed to be highly expressed in the spontaneous tumors and overall less expressed in the intestine of Andosan^™^-treated animals, we were not able to identify tumors in the examined gut section from the latter. Thus, a direct coupling of reduced legumain content to reduced tumor load and effect of Andosan^™^ on legumain expression within tumors, remains elusive in the examined intestines. Recently, it has been found that both Andosan^™^ as such and the polar high molecular weight fraction of it, inhibited production of legumain by a rat macrophage cell line (RAW264.7) as well as the activation of legumain proform [36]. It has been shown both that mice lacking legumain (asparaginyl endopeptidase) have normal levels of the major cytokines [41] and that cytokines do not alter legumain expression in epithelial cells [42]. Hence, the currently increased pro-inflammatory cytokines and others in serum should not be influenced by reduced intestinal (epithelial) legumain expression and vice versa in the Andosan^™^ treated mice.

This and other immuno-modulating effects such as increased cytokine production [43, 44], upregulation of adhesion molecules on leukocytes [45] and dendritic cell activation [46], are brought about by AbM—and probably also He and Gf—stimulation of monocytes, granulocytes and NK cells via TLR2, and probably dectin-1 and CD11b/18 [47,48]. In vitro Andosan^™^ stimulation of monocytic cells has shown increased expression of genes related to immune function [49], including the gene for IL-23 in the IL-12 family. This is in line with the current finding of an Andosan^™^-induced increase in the Th1 cytokine IL-12p70, which we have observed previously ex vivo in spleen cells harvested from Andosan^™^ treated Balb/c mice in the allergy model [8]. In vivo, oral intake of Andosan^™^ in a few patients with chronic HCV infection increased the expression in peripheral mononuclear leukocytes of genes related to tumor defense[10]. Probably β-glucans and other substances in mushrooms such as AbM act as danger signals and engage first the innate immune system, which then skews the adaptive immune system towards a Th1 type immune response. When studying the intestines by surface examination after administration or not of Andosan^™^, besides the tumors, no signs of necrosis or abnormal morphology were observed. This indicates that the mechanism of action for the Andosan^™^ effect in vivo in A/J *Min/+* mice is foremost protection against tumor development (tumorigenesis) in the intestines—possibly through indirect anti-tumor Th1 response of Andosan^™^—and to a lesser degree a direct cytotoxic effect on tumor cells (Fig 1). The animal study was terminated at 22 weeks of age for ethical reasons before any death from intestinal cancer occurred. The intestinal adenomas become adenocarcinomas in A/J *Min/+* mice at 30 weeks of age [27], where after the animals commence to die of cancer.

In the in vivo situation, the substances in Andosan^™^ will interact with the intestinal microbiota, which as a result may produce other biologically active metabolites that may affect the host. Since cereal β-glucans are found to alter gastrointestinal microbiota in pigs [50] and can ameliorate diseases through improvement of gut microbiota [51], such polysaccharides and other ingredients in Andosan^™^ may also have influenced the composition and activity of microbiota in the A/J *Min/+* mice in our experiments. Furthermore, substances in the mushroom extract may be absorbed across the intestinal wall by microfold (M) cells and DC, as shown for β-glucans in murine models [52]. Similar to β-glucan, also other biologically active mushroom-derived molecules may be transported further by DC to lymphocytes in gut-associated lymphoid tissue (GALT), example given Peyers’ patches, but also circulated in blood in the rodents [53].

The present observation in the mice of an increased pro-inflammatory response is in contrast to our findings with Andosan^™^ in humans. Previously, we have found reduced levels of pro-inflammatory serum cytokines after intake of Andosan^™^ in patients with the inflammatory bowel diseases, Crohn’s disease and ulcerative colitis (UC) [11]–the latter of which predisposing for colon cancer—and in patients with multiple myeloma [13]. In a very recent larger, placebo-controlled clinical study in UC patients we did also observe improved clinical effects of Andosan^™^ after 3 weeks [54]. Since the same dose of Andosan^™^ (60 ml/day) had also been used in the clinical multiple myeloma study for 7 weeks without side-effects [13], the extract is considered safe. Hence, to determine whether Andosan^™^ could be a safe, add-on treatment for patients with colorectal cancer, a clinical trial should be performed in such patients with relapse after surgery or who are on palliative treatment only.

## Supporting Information

S1 Text Hetland et al. Andosan, animal and apoptosis Raw Data. (XLSX)

## References

[1] Kerrigan RW. Agaricus subrufescens, a cultivated edible and medicinal mushroom, and its synonyms. Mycologia. 2005;97:12–24. 16389952 10.3852/mycologia.97.1.12

[2] Kawagishi H, Inagaki R, Kanao T, Mizuno T, Shimura K, Ito H, et al Fractionation and antitumor activity of the water-insoluble residue of Agaricus blazei fruiting bodies. Carbohydr Res. 1989;186(2):267–73. 2736561 10.1016/0008-6215(89)84040-6

[3] Itoh H, Ito H, Amano H, Noda H. Inhibitory action of a (1—>6)-beta-D-glucan-protein complex (F III-2-b) isolated from Agaricus blazei Murill ("himematsutake") on Meth A fibrosarcoma-bearing mice and its antitumor mechanism. Jpn J Pharmacol. 1994;66:265–271. 7869611 10.1254/jjp.66.265

[4] Kim SP, Nam SH, Friedman M. *Hericium* erinaceus (Lion’s Mane) Mushroom Extracts Inhibit Metastasis of Cancer Cells to the Lung in CT-26 Colon Cancer-Tansplanted Mice. J Agric Food Chem. 2013;61(20):, 4898–4904. 10.1021/jf400916c 23668749

[5] Suzuki I, Itani T, Ohno N, Oikawa S, Sato K, et al Antitumor activity of a polysaccharide fraction extracted from cultured fruiting bodies of Grifola frondosa. J Pharmacobiodyn. 1984 7;7(7):492–500. 6491867 10.1248/bpb1978.7.492

[6] Hetland G, Johnson E, Lyberg T, Bernardshaw S, Tryggestad AMA, Grinde B. Effects of the medicinal mushroom Agaricus blazei murill on immunity, infection and cancer. Scand J Immunol. 2008;68:363–370. 10.1111/j.1365-3083.2008.02156.x 18782264

[7] Bernardshaw S, Hetland G, Grinde B, Johnson E. An extract of the mushroom Agaricus blazei Murill protects against lethal septicemia in a mouse model of fecal peritonitis. Shock. 2006 4;25(4):420–5. 10.1097/01.shk.0000209526.58614.92 16670646

[8] Ellertsen LK, Hetland G. An extract of the medicinal mushroom Agaricus blazei Murill can protect against allergy. Clin Mol Allergy. 2009;7:6 (E pub). 10.1186/1476-7961-7-6 19416507 PMC2688003

[9] Takimoto H, Kato H, Kaneko M, Kumazawa Y. Amelioration of skewed Th1/Th2 balance in tumor-bearing and asthma-induced mice by oral administration of Agaricus blazei extracts. Immunopharmacol Immunotoxicol. 2008;30:747–760. 10.1080/08923970802279092 18720167

[10] Grinde B, Hetland G, Johnson E. Effects on gene expression and viral load of a medicinal extract from Agaricus blazei in patients with chronic hepatitis C infection. Int Immunopharmacol. 2006;6:1311–1314. 10.1016/j.intimp.2006.04.005 16782544

[11] Førland DT, Johnson E, Sæter L, Lyberg T, Lygren I, Hetland G. Effect of an Extract based on the medicinal mushroom Agaricus blazei Murill on expression of cytokines and calprotectin in patients with ulcerative colitis and Crohn’s disease. Scand J Immunol. 2011;73:66–75. 10.1111/j.1365-3083.2010.02477.x 21129005

[12] Johnson E, Førland DT, Sætre L, Bernardshaw SV, Lyberg T, Hetland G. Effect of an extract based on the medicinal mushroom Agaricus blazei Murill on release of cytokines, chemokines and leukocyte growth factors in human blood ex vivo and in vivo. Scand J Immunol. 2009;69:242–250. 10.1111/j.1365-3083.2008.02218.x 19281536

[13] Tangen JM, Tierens A, Caers J, Binsfeld M, Olstad OK, Trøseid AM et al Immunomodulatory effects of the Agaricus blazei Murrill-based mushroom extract AndoSan in patients with multiple myeloma undergoing high dose chemotherapy and autologous stem cell transplantation: a randomized, double blinded clinical study. Biomed Res Int. 2015;2015:718539 10.1155/2015/718539 25664323 PMC4312620

[14] Hetland G, Johnson E, Lyberg T, Kvalheim G. The Mushroom Agaricus blazei Murill Elicits Medicinal Effects on Tumor, Infection, Allergy, and Inflammation through Its Modulation of Innate Immunity and Amelioration of Th1/Th2 Imbalance and Inflammation. Adv Pharmacol Sci. 2011: 157015 10.1155/2011/157015 21912538 PMC3168293

[15] Takaku T, Kimura Y, Okuda H. Isolation of an antitumor compound from Agaricus blazei Murill and its mechanism of action. J Nutr. 2001 5;131(5):1409–13. 11340091 10.1093/jn/131.5.1409

[16] Pinheiro F, Faria RR, de Camargo JL, Spinardi-Barbisan AL, da Eira AF, Barbisan LF. Chemoprevention of preneoplastic liver foci development by dietary mushroom Agaricus blazei Murrill in the rat. Food Chem Toxicol. 2003 11;41(11):1543–50 12963007 10.1016/s0278-6915(03)00171-6

[17] Ziliotto L, Pinheiro F, Barbisan LF, Rodrigues MAM. Screening for in vitro and in vivo antitumor activities of the mushroom *Agaricus blazei*. Nutrition and Cancer, 2009;61(2), 245–250. 10.1080/01635580802395717 19235041

[18] http://www.oncolex.org.

[19] Hopkins MH, Flanders WD, Bostick RM. Associations of circulating inflammatory biomarkers with risk factors for colorectal cancer in colorectal adenoma patients. Biomark Insights. 2012;7:143–50. 10.4137/BMI.S10092 23170065 PMC3498968

[20] Fodde R. The APC gene in colorectal cancer. Eur J Cancer. 2002;38(7):867–71. 11978510 10.1016/s0959-8049(02)00040-0

[21] Half E, Bercovich D, Rozen P. Familial adenomatous polyposis. Orphanet J Rare Dis. 2009;4. 10.1186/1750-1172-4-22 PMC2772987 19822006

[22] Fearnhead NS, Britton MP, Bodmer WF. The ABC of APC. Hum Mol Genet. 2001;10(7):721–33. 11257105 10.1093/hmg/10.7.721

[23] Paulsen JE. Modulation by dietary factors in murine FAP models. Toxicology Letters. 2000;112:403–9. 10720759 10.1016/s0378-4274(99)00262-3

[24] van Es JH, Giles RH, Clevers HC. The many faces of the tumor suppressor gene APC. Exp Cell Res. 2001;264(1):126–34. 10.1006/excr.2000.5142 11237529

[25] Su LK, Kinzler KW, Vogelstein B, Preisinger AC, Moser AR, Luongo C, et al Multiple Intestinal Neoplasia Caused by a Mutation in the Murine Homolog of the Apc Gene. Science. 1992;256(5057):668–70. 1350108 10.1126/science.1350108

[26] Fodde R, Smits R. Disease model: familial adenomatous polyposis. Trends Mol Med. 2001;7(8):369–73. 11516998 10.1016/s1471-4914(01)02050-0

[27] Sodring M, Gunnes G, Paulsen JE. Spontaneous initiation, promotion, and progression of colorectal cancer in the novel A/J Min/+ mouse. Int J Cancer. 2016 4 15;138(8):1936–46. 10.1002/ijc.29928 26566853

[28] Liu C, Sun C, Huang H, Janda K, Edgington T. Overexpressionof legumain in tumors is significant for invasion/metastasis and a candidate enzymatic target for prodrug therapy. Cancer Res. 2003;63:2957–2964. 12782603

[29] Miller G, Matthews SP, Reinheckel T, Fleming S, Watts C. Asparagine endopeptidase is required for normal kidney physiology and homeostasis. FASEB J. 2011;25:1606–1617. 10.1096/fj.10-172312 21292981

[30] Murthy RV, Arbman G, Gao J, Roodman GD, Sun XF. Legumain expression in relation to clinicopathologic and biological variables in colorectal cancer. Clin Cancer Res. 2005;11:2293–2299. 10.1158/1078-0432.CCR-04-1642 15788679

[31] Gawenda J, Traub F, Luck HJ, Kreipe H, von Wasielewski R. Legumain expression as a prognostic factor in breast cancer patients. Breast Cancer Res Treat. 2007;102:1–6. 10.1007/s10549-006-9311-z 17028987

[32] Liu Y, Bajjuri K M, Liu C, Sinha SC. Targeting Cell Surface Alpha(v)beta(3) Integrins Increases Therapeutic Efficacies of a Legumain Protease-Activated Auristatin Prodrug. Mol Pharm. 2012 1 1; 9(1): 168–175. NIHMSID: NIHMS340114. 10.1021/mp200434n 22044266 PMC3277864

[33] Morita Y, Araki H, Sugimoto T, Takeuchi K, Yamane T, Maeda T, et al Legumain/asparaginyl endopeptidase controls extracellular matrix remodeling through the degradation of fibronectin in mouse renal proximal tubular cells. FEBS Lett. 2007;581:1417–1424. 10.1016/j.febslet.2007.02.064 17350006

[34] Lin Y, Wei C, Liu Y, Qiu Y, Liu C, Guo F. Selective ablation of tumor-associated macrophages suppresses metastasis and angiogenesis. Cancer Sci. 2013 9;104(9):1217–25. 10.1111/cas.12202 23691974 PMC3766435

[35] Haugen MH, Boye K, Nesland JM, Pettersen SJ, Egeland EV, Tamhane T et al High expression of the cysteine proteinase legumain in colorectal cancer—implications for therapeutic targeting. Eur J Cancer. 2015 1;51(1):9–17. 10.1016/j.ejca.2014.10.020 25466510

[36] Berven L, Karppinen P, Hetland G, Samuelsen AB. The polar high molecular weight fraction of the Agaricus blazei Murill extract, AndoSan™, reduces the activity of the tumor-associated protease, legumain, in RAW 264.7 cells. J Med Food. 2015 4;18(4):429–38. 10.1089/jmf.2014.0018 25136950

[37] Paulsen JE, Elvsaas IK, Steffensen IL, Alexander J. A fish oil derived concentrate enriched in eicosapentaenoic and docosahexaenoic acid as ethyl ester suppresses the formation and growth of intestinal polyps in the Min mouse. Carcinogenesis. 1997 10;18(10):1905–10 9363998 10.1093/carcin/18.10.1905

[38] Holson RR, Freshwater L, Maurissen JP, Moser VC, Phang W. Statistical issues and techniques appropriate for developmental neurotoxicity testing: a report from the ILSI Research Foundation/Risk Science Institute expert working group on neurodevelopmental endpoints. Neurotoxicol Teratol. 2008;30(4):326–48. 10.1016/j.ntt.2007.06.001 17681748

[39] Masuda Y, Inoue H, Ohta H, Miyake A, Konishi M, Nanba H. Oral administration of soluble β-glucans extracted from Grifola frondosa induces systemic antitumor immune response and decreases immunosuppression in tumor-bearing mice. Int J Cancer. 2013 7;133(1):108–19. 10.1002/ijc.27999 23280601

[40] Gao L, Sun Y, Chen C, Xi Y, Wang J, Wang Z. Primary mechanism of apoptosis induction in a leukemia cell line by fraction FA-2-b-ss prepared from the mushroom Agaricus blazei Murill. Braz J Med Biol Res. 2007 11;40(11):1545–55. 17934651 10.1590/s0100-879x2007001100015

[41] Chan CB, Abe M, Hashimoto N, Hao C, Williams IR, et al Mice lacking asparaginyl endopeptidase develop disorders resembling hemophagocytic syndrome. Proc Natl Acad Sci U S A. 2009 1 13;106(2):468–73. Epub 2008 Dec 23. 10.1073/pnas.0809824105 19106291 PMC2626726

[42] Luo Y, Zhou H, Krueger J, Kaplan C, Lee SH, Dolman C, et al Targeting tumor-associated macrophages as a novel strategy against breast cancer. J Clin Invest. 2006 8;116(8):2132–2141. 10.1172/JCI27648 16862213 PMC1513049

[43] Sorimachi K, Akimoto K, Ikehara Y, Inafuku K, Okubo A, Yamazaki S. Secretion of TNF-alpha, IL-8 and nitric oxide by macrophages activated with *Agaricus blazei* Murill fractions in vitro. Cell Struct Funct. 2001; 26:103–108. 11482452 10.1247/csf.26.103

[44] Førland DT, Johnson E, Tryggestad AMA, Lyberg T, and Hetland G. An extract based on the medicinal mushroom Agaricus blazei Murill stimulates monocyte-derived dendritic cells to cytokine and chemokine production in vitro. Cytokine. 2010; 49 (3): 245–250. 10.1016/j.cyto.2009.09.002 20036142

[45] Bernardshaw S, Lyberg T, Hetland G, Johnson E. Effect of an extract of the mushroom Agaricus blazei Murill on expression of adhesion molecules and production of reactive oxygen species in monocytes and granulocytes in human whole blood ex vivo. APMIS. 2007; 115(6):719–25. 10.1111/j.1600-0463.2007.apm_619.x 17550380

[46] Tangen J-M, Tryggestad AMA and Hetland G. Stimulation of human monocytic cells by the medicinal mushroom Agaricus blazei Murill induces expression of cell surface markers associated with activation and antigen presentation. Applied Scientific Reports. 2014; 1(1): 1.

[47] Gatner BN, Simmons RM, Canavera SJ, Akira S, Underhill DM.Collaborative induction of inflammatory responses by dectin 1 and Toll-like receptor 2. J Exp Med. 2003; 5 5;197(9):1107–17. 10.1084/jem.20021787 12719479 PMC2193968

[48] Vetvicka V, Thornton BP, Ross GD. Soluble β-glucan polysaccharide binding to the lectin site of neutrophil or natural killer cell complement receptor type 3 (CD11b/CD18) generates a primed state of the receptor capable of mediating cytotoxicity of iC3b-opsonized target cells. J Clin Invest. 1996;98:50–61. 10.1172/JCI118777 8690804 PMC507400

[49] Ellertsen LK, Hetland G, Johnson E, and Grinde B. Effect of a medicinal extract from Agaricus blazei Murill on gene expression in a human monocyte cell line as examined by microarrays and immuno assays. Int Immunopharmacol. 2006; 6 (2): 133–143. 10.1016/j.intimp.2005.07.007 16399618

[50] Metzler-Zebeli BU, Zijlstra RT, Mosenthin R., and Gänzle MG. Dietary calciumphosphate content and oat β-glucan influence gastrointestinal microbiota, butyrate-producing bacteria and butyrate fermentation in weaned pigs. FEMS Microbiol Ecol. 2011;75:402–413 10.1111/j.1574-6941.2010.01017.x 21166688

[51] Yoo JY, Kim SS. Probiotics and Prebiotics: Present Status and future perspectives on metabolic disorders. Nutrients. 2016 3 18;8(3). Pii: E173. 10.3390/nu8030173 PMC4808900 26999199

[52] De Jesus M, Ostroff GR, Levitz SM, Bartling TR, Mantis NJ. A population of Langerin-positive dendritic cells in murine Peyer's patches involved in sampling β-glucan microparticles. PLoS One. 2014 3 14;9(3):e91002 eCollection 2014. 10.1371/journal.pone.0091002 24632738 PMC3954581

[53] Ikuzawa M, Matsunaga K, Nishiyama S, Nakajima S, Kobayashi Y, Andoh T, et al Fate and distribution of an antitumor protein-bound polysaccharide PSK (Krestin). Int J Immunopharmacol. 1988;10(4):415–23. 3170055 10.1016/0192-0561(88)90128-2

[54] Therkelsen SP, Hetland G, Lyberg T, Lygren I, Johnson E. Effect of a Medicinal Agaricus blazei Murill-based Mushroom Extract, AndoSanTM, on Symptoms, Fatigue and Quality of Life in Patients with Ulcerative Colitis in a Randomized Single-Blinded Placebo Controlled Study. PLOS One. 2016 3 2; 10.1371/journal.pone.0150191 PMC4944955 27415795

